# Inhibition of cancerous properties of triple-negative MDA-MB-436 cells by targeting the K^+^ voltage-dependent Kv2.1 channel

**DOI:** 10.1007/s13105-025-01134-2

**Published:** 2025-11-04

**Authors:** Rita Canella, Anna Terrazzan, Francesca P. Carbone, Silvia Grassilli, Carlo M. Bergamini, Valeria Bertagnolo, Federica Brugnoli, Pietro Ancona, Nicoletta Bianchi

**Affiliations:** 1 Department of Neuroscience and Rehabilitation, University of Ferrara, Ferrara, Italy; 2 Department of Translational Medicine, University of Ferrara, Ferrara, Italy; 3 Genomics Core Facility, Centre of New Technologies, University of Warsaw, Warsaw, Poland; 4 Department of Environmental Sciences and Prevention, University of Ferrara, Ferrara, Italy; 5 Laboratory for Advanced Therapeutic Technologies, University of Ferrara, Ferrara, Italy

**Keywords:** Potassium voltage-dependent channel, Kv2.1 channel, KCNB1, Breast cancer cells, MDA-MB-436, Drofenine, Cell migration

## Abstract

We have investigated the involvement of K^+^ channels in generating the membrane current in MDA-MB-436 cells, a model of triple-negative breast cancer (TNBC). The membrane current is strongly influenced by the opening of voltage-dependent channels insensitive to the nonspecific K^+^ channel inhibitor 4-aminopyridine (4-AP). Using the cell patch clamp technique, we observed a significant decrease in membrane current after exposure to the generic K^+^ channel inhibitor tetraethylammonium chloride (TEA-Cl), indicating that K^+^ ions contribute to the overall membrane current through K^+^ channels that are insensitive to 4-AP but TEA-Cl-sensitive. RNA-sequencing analysis identified the Big Potassium (BK or Maxi-K or KCa1.1, encoded by *KCNMA1*) and the Kv2.1 (encoded by *KCNB1*) channels as putative candidates, both of which are involved in cancer cell proliferation and migration. Iberiotoxin, a specific inhibitor of BK channels, did not affect the total membrane current, just as CdCl₂ did, a potent inhibitor of Ca^2^⁺ channels involved in BK activation. Using selective inhibitors, stromatoxin and drofenine, we demonstrated that the Kv2.1 channel contributes to the membrane current in MDA-MB-436 cells. Furthermore, drofenine inhibited cell migration as measured by the xCELLigence Real-Time Cell Analyzer System and induced apoptosis. Single-cell analysis revealed that the Kv2.1 channel is expressed in both normal and cancerous tissues, with significant upregulation in brain metastases. This raises the possibility that Kv2.1 could be explored as a potential therapeutic target for controlling advanced stages of the neoplasia.

## Background

In the last decade, considerable attention has been paid to the role of voltage-gated K^+^ channels in the onset, development, spread, and metastatic dissemination of cancer [1].

Among them, the Big Potassium channel (BK or Maxi-K or KCa1.1) is a voltage-gated channel present in nearly every cell type [2]. Its opening probability rises with increasing intracellular calcium concentration, and the blockage of calcium channels affects the amplitude of the current [3]. BK is expressed in different cellular compartments and its activity is modulated by various post-translational modifications and by interactions with several proteins to regulate cell division and migration [4–6]. Due to their altered functionality, especially under hypoxic conditions, BK channels are druggable targets, as demonstrated in mouse xenograft models of hepatocellular carcinoma [7]. BK members represent also specific targets for immunotherapy [8]. Focusing on breast cancer, screening of panels of cell lines by next-generation sequencing identified sets of K^+^ channels associated with a specific phenotype [9] or a malignancy related process [10]. In particular, in triple-negative breast cancer (TNBC) cells, membrane potential is usually depolarized and BK channels are inactive. Their reactivation promotes the induction of membrane hyperpolarization and cell apoptosis [9], while in estrogen receptor-positive tumors, responsive to tamoxifen, these channels influence survival in knock-out mice [11]. Especially in TNBC, investigations revealed the role of certain K^+^ channels in cancer evolution and metastasis [12–15] as regulator of cellular pathways [16]. Recent findings described the ability of voltage-gated K + channels to rewire cancer cell metabolism [17], interfering with cell division and motility [4].

Many studies also demonstrated a strong link between Kv channels and cancer development [18]. Kv are the most complex K^+^ channels, regulating cellular excitability and participating in cancer development and progression by controlling cell proliferation, volume, apoptosis, and migration [18–21]. Changes in K^+^ permeability due to Kv channels can hyperpolarize the cell at the end of the G1 phase of cell cycle and cause depolarization at the G2/M, influencing cell growth. Cell volume regulation is also intrinsically linked to membrane potential changes, with hyperpolarization favoring cell shrinkage, which in turn can initiate Ca^2+^ oscillations, crucial for proliferation [18].

Among Kv channels, Kv2.1 (encoded by *KCNB1* gene) displays a role in various tumors including gastric [22] and endometrial cancer [23], in which Kv2.1 forms complexes with other homo-multimer functional channels such as Kv9.3 that modulate the electrophysiological properties of excitable cells, exerting a potential oncogenic role [24]. A recent investigation of *KCNB1* gene polymorphisms found that some variants significantly associate with treatment response in colorectal cancer, helping treatment strategies personalization and disease kinetics monitoring, during chemotherapy [24, 25]. Furthermore, there is an involvement of this voltage-gated potassium channel in medulloblastoma where authors found a relationship between Kv2.1 channels and heme oxygenase-1, regulating the susceptibility to apoptosis in neurons and tumor cells [26]. In addition, Kv2.1 is interestingly associated to growth and metastasis in prostate tumors [27] and to migration in breast cancer cells, in which it exerts regulatory functions by mechanisms unrelated to membrane hyperpolarization and Ca^2+^ influx [28].

The clear interest in potential therapeutic applications identifies a wide range of K^+^ channels as possible molecular targets. In breast cancer, researchers investigated this perspective [29–31], showing that specific K^+^ channels’ inhibitors can reduce *in vitro* cell migration, if used as single drug [32], e.g. chloroquine, blocking Kv10.1, or in combination with other compounds, such as astemizole and gefitinib, active against K^+^ channel ether-à-go-go-1 [33].

Notably, distinct K^+^ channels might play a role in cancer processes in specific cell lines. In a previous study, we investigated by patch-clamp, the impact of a transglutaminase type 2 (TG2) inhibitor on membrane current in three TNBC cell lines (MDA-MB-231, MDA-MB-436 and MDA-MB-468 cells), showing the potential engagement of different K^+^ voltage-dependent channels [15]. We demonstrated that Kv10.1 plays a pivotal role only in MDA-MB-231 cells, leaving unresolved the question regarding MDA-MB-436 cells, which share the same TNBC phenotype, but exhibit different malignant characteristics.

Considering their relevance, and the fact that their specific role in cancer is complex and not fully understood, complicated by the variety of cell types, tumor stages, and auxiliary proteins involved, in the present study we further investigated K^+^ channels using patch-clamp and unspecific/specific inhibitors, to define which was predominant and responsible for the observed current in MDA-MB-436 cells.

## Methods

### Cell cultures

MDA-MB-436 (CVCL_0623) TNBC cells were obtained from the American Type Culture Collection (Rockville, MD, USA).

Cells were cultured in Dulbecco's Modified Eagle Medium high glucose (Gibco™, Thermo Fisher Scientific, Monza, Italy) supplemented with 10% fetal bovine serum (FBS, Thermo Fisher Scientific, Monza, Italy), 2 mM glutamine, 50 U/mL penicillin, and 50 μg/mL streptomycin (Merck Life Science), and detached by trypsinization to be subjected to specific tests.

They were tested monthly for mycoplasma. The expression of genes defining the phenotype of MDA-MB-436 cells was validated using an RNA-sequencing dataset reported in our previous publication [34].

### Patch-clamp technique and compounds employed

Patch pipettes were pulled from glass capillaries with 1.0 mm outer diameter using a micropipette puller (NARISHIGE Instruments, Japan, mod PP-830), fire-polished (tip resistance between 2 and 5 MOhm) and filled with an intracellular solution to characterize the overall membrane current response. This had the following composition: 145 mM KCl, 1 mM MgCl_2_, 10 mM HEPES, and 5 mM EGTA; pH = 7.3 adjusted with KOH; while the perfusion solution was constituted as follows: 145 mM NaCl, 1.8 mM CaCl_2_, 1 mM MgCl_2_, 5.4 mM KCl, 10 mM glucose, and 10 HEPES; pH = 7.35 adjusted with NaOH.

In experiments aimed at eliminating the K^+^ current, we substituted KCl with CsCl (130 mM) in the intracellular solution adjusting to the pH = 7.3 with tetraethylammonium hydroxide (TEA-OH, Sigma-Aldrich, St. Louis, Missouri, US). If necessary, osmolality was adjusted with sucrose to obtain values between 300 and 310 mOsm/Kg [35].

We employed 10 mM TEA-Cl (Sigma-Aldrich) as a generic inhibitor of K^+^ channels [36] and 200 μM cadmium chloride (CdCl_2_, Sigma-Aldrich) to interfere with KCa channels; 100 nM Iberiotoxin (IbTx, Sigma-Aldrich) specific blocker of BK channels [37] dissolved in water and added to the perfusion solution [38, 39].

Other assayed compounds were TG2 inhibitor LDN27219 (ZEDIRA GmbH, Darmstadt, Germany) dissolved in dimethyl sulfoxide (DMSO) and added to the intracellular solution up to the final concentration of 9 μM; stromatoxin (ScTx1; Alomone Labs, Jerusalem BioPark, Jerusalem, Israel) dissolved in buffered solution and added to the perfusion solution up to the final concentration of 600 nM [27]; drofenine (Dfe; Sigma-Aldrich) dissolved in DMSO and added to the perfusion solution to achieve the final concentration of 10 μM [40].

Cells were observed through a TV monitor connected to a contrast-enhanced video camera (T.I.L.L. Photonics, Planegg, Germany) coupled to an inverted microscope (Olympus IMT-2, Tokyo, Japan) equipped with a 40 × Hoffman-modulation contrast objective.

Whole-cell currents were elicited by voltage-clamp pulses between −30 mV and + 70 mV in 20 mV steps from a holding potential of −30 mV.

Before recording, cells exhibited high membrane resistance (> 1 GOhm) at −70 mV. These values indicate the quality of the seal and that no major conductances, such as hyperpolarization-activated currents were significantly active. Access resistance ranged between 8 and 22 MOhm. The voltage protocol and data acquisition were performed with Digidata card 1322 A and the pClamp software package (version 9.2). The currents were recorded with a commercial patch-clamp amplifier (EPC-7; Consumer E-List, Darmstadt, Germany); the recordings were filtered at 5 kHz and acquired at 10 kHz [41].

### Real-time assays of cell migration

MDA-MB-436 cells were collected, and their migration ability was analyzed by xCELLigence RTCA system (Real-Time Cell Analyzer System, Acea Biosciences Inc., San Diego, CA, USA), as previously reported [42]. About 4 × 10^5^ cells/well were seeded onto the top chambers of CIM-16 plates in DMEM medium without FBS and the bottom chambers were filled with medium containing 5% serum, used as chemoattractant. Signal detection was recorded every 15 min for 24 h. We expressed impedance values as Cell Index (CI), and values greater than 0.1 were taken as relevant. The kinetic of cell migration was also quantified by calculating the slope, a value indicating the steepness, inclination, gradient, and changing rate of the CI curves over time.

### Cell treatments, antiproliferative and apoptotic assays

About 120 × 10^5^ cells/mL were seeded in 2 mL well-plates and treated with Dfe 10–50–100 μM or with the vehicle for 48 h. Apoptotic effects were evaluated using the Muse® Annexin V and Dead Cell Kit (Prodotti Gianni, Milan, Italy) and MUSE cell analyzer [43]. The samples resuspended in medium with 10% FBS and containing almost 5 × 10^5^ cells/mL were diluted 1:2 with the kit solution, incubated for 20 min at room temperature, and finally analyzed.

### Data analysis and statistical procedures

Data reported in the text and figures are presented as mean ± SEM or SD, as specified in the legends to figures. The control-treated comparisons were made with GraphPad Prism v.5, and the significance of *P* values were reported in the legends of the figures (two-way ANOVA, Bonferroni post hoc test; significant differences for *P* < 0.05). The comparisons between the groups were made using GraphPad Prism v.5, and the significance of *P* values was reported in the legends of the specific figures.

Statistical analysis of cell migration was performed by using the 2-tailed Student’s *t* test for unpaired data calculated with the GraphPad Prism 6.0 statistical package (GraphPad Software, San Diego, CA, USA). Statistical significance in the apoptosis assays was calculated using a two-tailed unpaired *t*-test with type 2 variance (equal variance assumed).

### Single-cell RNA-sequencing analysis

We used 16 profiles of single-cell RNA-sequencing downloaded from GEO (GSE161529, GSE245601) databases, including primary breast cancer biopsies from different phenotypes: human epidermal growth factor (HER2)-enriched (n = 4), ER^+^ (n = 4), TNBC (n = 4), and healthy tissues (n = 4). Raw data quality was inspected using MultiQC [44], and fastq files were aligned to the GRCh38genome reference using Genomic Cell Ranger v8.0.1 software [45]. CellBender v0.3.1 was used to filter out empty droplets [46]. The downstream analysis was carried out by Scanpy v1.10.3 [47] and doublets were removed using the integrated Scrublet package. Also, cells expressing < 1000 total counts have been removed.

A modified Z-score approach was employed to identify the outliers. Data points deviating from the median by more than n-median absolute deviations were excluded as well as cells expressing more than 5% of mitochondrial counts. Normalization of the raw counts was performed to a total count of 10.000 per cell, subsequently log-transformed using algorithm plus one (log1p). After a principal component analysis, data was integrated by Harmony Scanpy.

## Results

### Identification of * K*^+^*channels * involved in the current profile of MDA-MB-436 cells using * unspecific * and specific * inhibitors*

In our previous work [15], we demonstrated that, unlike other TNBC cell lines, the total membrane current in MDA-MB-436 cells was influenced by voltage-dependent K⁺ channels that were insensitive to the non-specific K^+^ channel inhibitor 4-AP. Based on these observations and knowing that the membrane current depends on the contribution by several types of ions (K^+^, Cl^−^, Na^+^, Ca^2+^, etc.), we tried to understand if K^+^ ions are significantly involved in its generation.

**Fig. 1:**
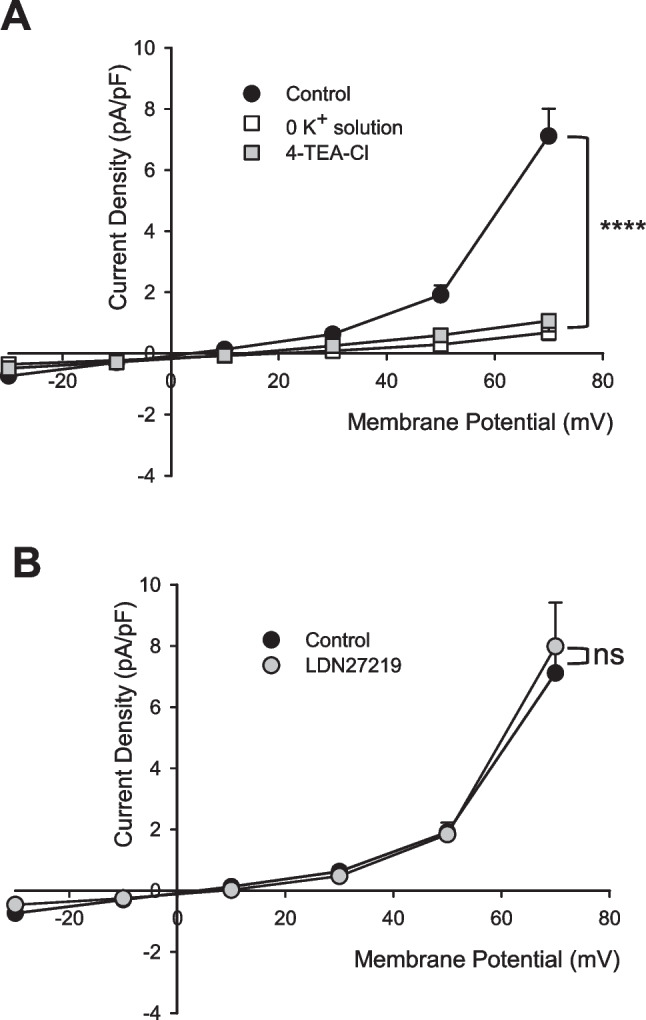
Membrane current sustained by K^+^ ions and channel inhibited by TEA-Cl. (A) The current–voltage relationship was analyzed in control cells (n = 14), in the absence of K^+^ ions (marked 0 K^+^) in the intracellular solution (n = 7), and after treatment with TEA-Cl (n = 6). (B) The current–voltage relationship of control cells (n = 14) and LDN27219 treated cells (n = 6). The results were expressed as average ± SEM. pA⋅pF − 1 (picoAmpere/picoFarad); ****, *p* < 0.0001, evaluated using two way ANOVA; ns = not significant

Firstly, we measured the current–voltage relationship (I-V) in the presence and absence of K^+^ ions in the intracellular solution (Fig. 1A). In the latter condition, the ions contributing to the current are Cl^−^. The difference between the curves of control and 0 K^+^ was statistically significant, demonstrating a strong dependency on K^+^. Furthermore, after the addition of TEA-Cl, a K^+^ current generic inhibitor, we observed a substantial decrease in current density (Fig. 1A). These results indicated that the contribution by K^+^ ions is preponderant, involving 4-AP insensitive but TEA-Cl sensitive K^+^ channels. In addition, since we had already demonstrated that the functionality of the Kv10.1 channel is modulated by TG2, a multifunctional enzyme with both transamidase catalytic activity, in its open form, and G-protein-like activity in its closed conformation [15], we used LDN27219, a TG2specific inhibitor [48]. The data shown in Fig. 1B further confirmed that the Kv10.1 channel appears to be neither functional nor responsive to TG2 modulation in MDA-MB-436 cells, suggesting that a different K⁺ channel may be responsible.

**Fig. 2:**
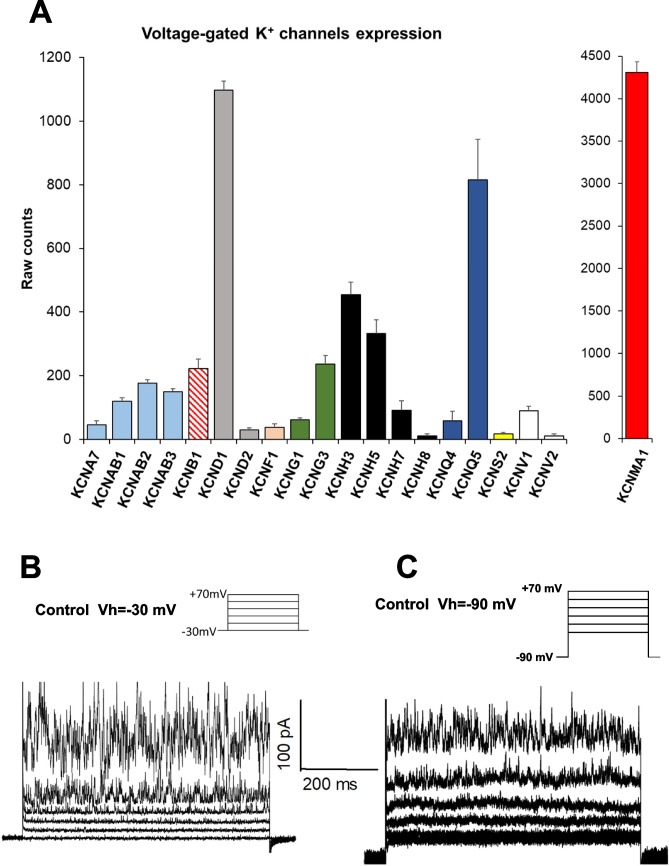
K^+^ channels and current profile in MDA-MB-436 cells. (A) Expression of K^+^ channels in MDA-MB-436 cells graphed as average ± SD of raw counts from RNA-sequencing obtained analyzing three different samples. In the left panel, transcripts for voltage-dependent K^+^ channels, in right panel, those for KCa1.1 BK channel. (B and C) Representative families of total recording currents in the control condition. Each line represents the current values obtained at each time step while the cell is held at the corresponding potential level indicated in the protocol insert. The axes have been omitted and replaced by calibration. The holding potential for recording in panel B is −30 mV, while the holding potential in panel C is −90 mV. No indication of inactivation removal is present

Based on a transcriptomic analysis [34], six genes coding voltage-dependent K^+^ channels are expressed at high levels in MDA-MB-436 cells, as shown in Fig. 2A. They are *KCNB1* coding the Kv2.1 channel, *KCND1* for the Kv4.1, *KCNG3* for the Kv6.3, *KCNH3* for the Kv12.2, *KCNH5* for the Kv10.2, and *KCNQ5* encoding the Kv7.5.

We excluded the possible involvement of the Kv4.1 [49, 50], and Kv12.2 channels [49, 51] because they are inactivating channels [49], while our recording traces showed no inactivation for the duration of the step (approximately 700 ms), neither when the holding potential (Vh) was −30 mV (Fig. 2B) nor −90 mV (Fig. 2C). In fact, the sejour at −90 mV would have removed the channel inactivation such as Kv4.1 and Kv12.2, producing current traces showing a peak at the beginning of the voltage step. However, the traces recorded with Vh = −90 mV were very similar to those obtained with Vh = −30 mV. As Vh = −90 mV made the seal unstable because it was so far from physiological values in these cells, we continued our experiments maintaining Vh at −30 mV.

This makes the Kv2.1 channel a good candidate for our analysis, because the literature indicates that it does not show fast [49], but a slow inactivation with time constant of 4 to 7 s [52]. Concerning the Kv6.3 channel, it results in electrical silence [49, 53]. Finally, we also excluded the possible involvement of the Kv7.5 channel, as it is inhibited by TEA-Cl only at concentrations above 30 mM, and of the Kv10.2 channel, which is insensitive to TEA-Cl [36, 49]. We also quantified the mRNA of the BK channel (*KCNMA1*), which has a unique characteristic among KCa channels: it possesses both voltage and calcium sensors. This means its open probability is significantly influenced by both membrane depolarization and intracellular calcium [54] and we detected appreciable levels of the transcripts with 4310 ± 120 raw counts, as shown in the right panel of Fig. 2A.

In the literature, Wulff et al. [36] reported eight types of K^+^ channels insensitive to 4-AP and sensitive to TEA-Cl. After merging that list with a series of channels we found to be expressed in MDA-MB-436 cells (Fig. 2A), the KCa1.1 and Kv2.1 were present in both and emerged as the most interesting.

**Fig. 3:**
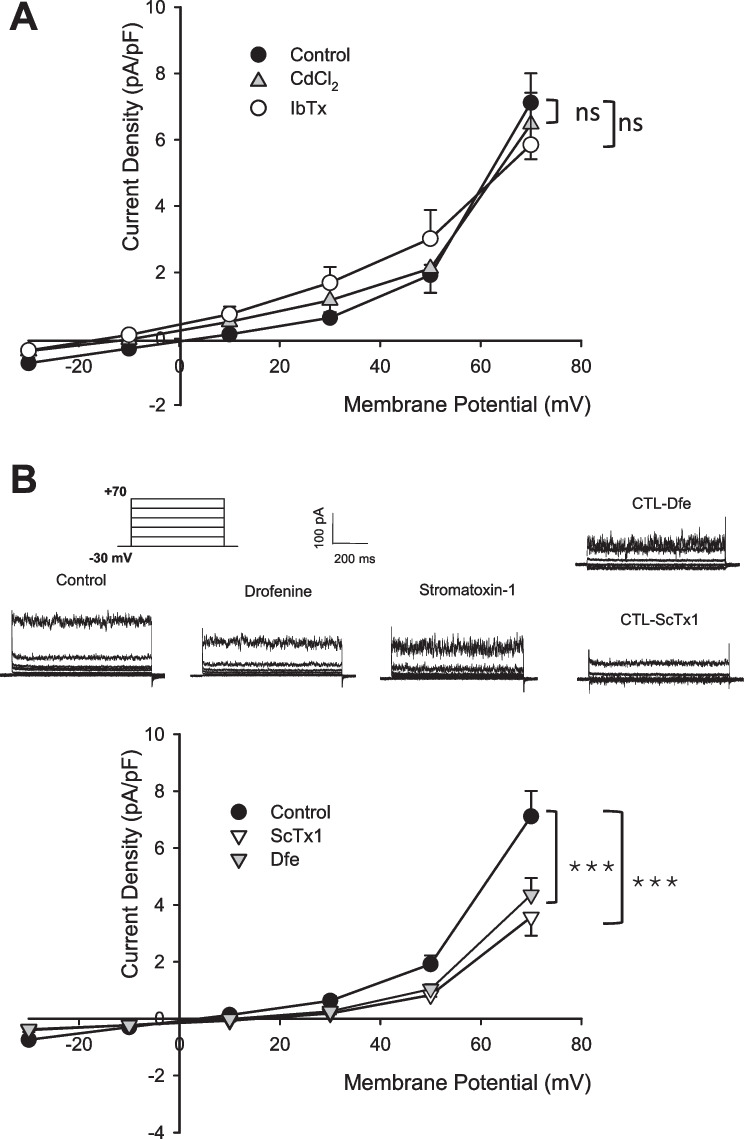
Effects of K^+^ channel inhibitors. (A) Control (n = 14) and IbTx (n = 5) and CdCl_2_ (n = 5) treated cells. (B) Control (n = 14), ScTx1 (n = 5) or Dfe (n = 10) treated cells. The results were expressed as average ± SEM. pA⋅pF − 1 (picoAmpere/picoFarad); ***, *p* < 0.001, evaluated using two way ANOVA; ns = not significant

The first is a Ca^2+^-gated BK channel sensitive to CdCl_2_, a powerful inhibitor of Ca^2+^ channels [55, 56]. It is possible to influence the conductance of this channel by blocking the influx of Ca^2+^ that occurs through voltage-gated Ca^2+^ channels. In fact, as reported by Hille [19], the entry of calcium into the cell increases the Ca^2+^ concentration near the inner side of the membrane, causing an increase in the conductance of BK channels. Treating the cells with CdCl_2_, a powerful inhibitor of Ca^2+^ channels [55, 56] it is possible to reduce the BK conductance [38, 57]. When 200 μM CdCl_2_ was applied to the perfusion solution, we observed no effect on the total membrane current (Fig. 3A), indicating that, despite high levels of mRNA expression for the KCa1.1 channel, it was not functional. Our observation was strengthened by the application in the perfusion solution of IbTx, a specific blocker of BK channels [35]. Figure 3A shows that in this case, too, the current is not statistically different from control.

Kv2.1 channels are selectively inhibited by ScTx1, a toxin identified in the venom of the African tarantula *Stromatopelma calceatum* [27, 58, 59]. Biologically effective concentrations extend up to 600 nM. We used 600 nM ScTx1 because, while this dose is elevated compared to concentrations commonly used, it is considered biologically acceptable for this channel [27]. We believe this was necessary because the abundant extracellular matrix in MDA-MB-436 cells may have hindered the access of the compound to the plasma membrane at lower concentrations, especially considering the relatively large size of ScTx1 (composed of 34 amino acids).

Moreover 10 μM Dfe, an antimuscarinic antispasmodic drug active on TRPV3 and Kv2.1 channels was tested [40]. We exclude the involvement of TRPV3 channels as their effective activation doses are 250/500 µM [60]. In addition, the kinetics would be different, as the time scale for observing TRPV3 channel currents is the order of seconds. Regarding M1 muscarinic receptors, their activation reduces the potassium current in cells, so their inhibition by Dfe would be expected to cause an increase in total current, whereas we observe a decrease. Both these inhibitors induced a very significant decrease in the membrane current compared to control (Fig. 3B). We provide our interpretation of this in the Discussion section. This evidence demonstrated that the Kv2.1 channel is active in the generation of the membrane current with a substantial contribution. However, after both Dfe and ScTx treatments, a residual current of about 4 pA/pF for Dfe treatment and 3.4 pA/pF for ScTx treatment at + 70 mV remains, as shown in Fig. 3B. Part of this current is due to Cl^−^ ion movement and leakage current, as demonstrated in Fig. 1A in the absence of K^+^, and to a partial effect of inhibitors; nevertheless, it is not possible to exclude some little contribution from other K^+^, heteromers of Kv2.1 or cationic channels. The insert shows example current traces of different control, Dfe and ScTx treated cells; additionally, it illustrates the current eliminated by the inhibitors, derived from the subtraction of traces from control traces, representing the current resulting from Kv2.1 channel activation.

### Dfe inhibits motility and induces apoptosis in MDA-MB-436 cells

Since the membrane current in MDA-MB-436 cells is sustained by the Kv2.1 channel, which can be blocked by Dfe, we investigated whether targeting this channel could affect their cancerous properties, such as migration ability. Indeed, among voltage-dependent channels, Kv2.1 has been linked to cell cycle progression and migration [61].

**Fig. 4:**
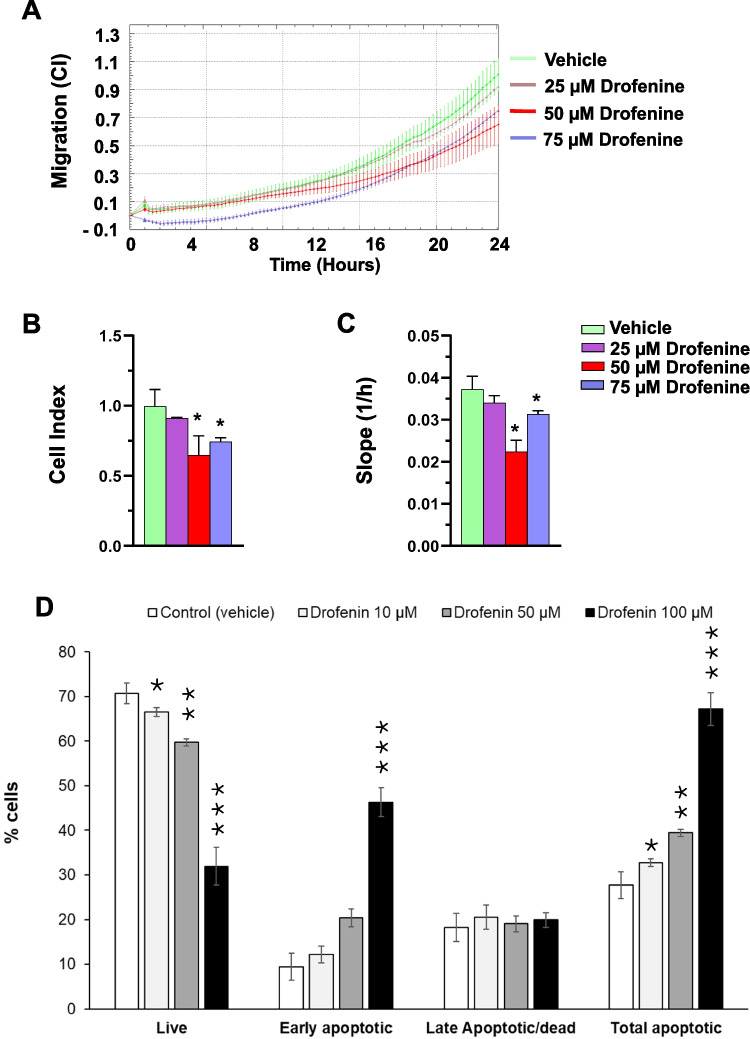
Effects of Dfe on cellular functions. (A) Cell dynamic monitoring for 24 h. In Green, control samples were treated only with the vehicle, while in Lilac, Red, and Violet, samples were treated with 25, 50, and 75 μM of Dfe. (B) CI (cell index) and (C) slope analysis, considering the steepness, inclination, gradient, and changing rate of the CI curves over time. The results were graphed as average ± SD of three independent experiments. The statistical analysis reported in the slope histogram was performed by a two-tailed Student’s *t*-test for unpaired data analyzed by GraphPad Prism 6.0 statistical package (GraphPad Software, San Diego, CA, USA). *, indicated *p* < 0.05. (D) Dfe-induced apoptosis in MDA-MB-436 cells. Percentage of apoptotic cells evaluated after treatment with the indicated concentrations of Dfe. Different stages of apoptosis: Live, Early Apoptosis, Late Apoptosis, and Total apoptotic cells were reported as average ± SD of three independent experiments. Statistical significance was assessed using a two-tailed unpaired *t*-test with type 2 variance (equal variance assumed). *, indicated *p* < 0.05; **, indicated *p* < 0.01; ***, indicated *p* < 0.001

Exposing these TNBC cells to several concentrations of Dfe for 24 h, we monitored their motility in the xCELLigence RTCA system. We showed the cell index (CI) curves in Fig. 4A, the CI values derived from the average of all replicates measured after 24 h in Fig. 4B, and the migration rate (slope) throughout the entire assay duration in Fig. 4C, which represents the change in CI over time. The motility of MDA-MB-436 cells in the presence of Dfe was markedly reduced compared to the vehicle-treated cells, particularly at the 50 μM concentration, which decreased migration by approximately 40% (Fig. 4B and 4 C), likely through inhibition of the Kv2.1 channel. No statistically significant differences in cell motility were observed between 50 and 75 μM Dfe over time.

Further to prevent cells from moving, we checked whether Dfe induced cell death in MDA-MB-436 cells. We evaluated the apoptotic effects of this compound after 48 h treatment with the vehicle (control) or with 10, 50 100 μM Dfe. Total apoptotic cells were significantly increased (Fig. 4D), reaching 39.5 ± 0.82 and 67.2 ± 3.68 at 50 and 100 μM Dfe, respectively, indicating the potential to drive TNBC cells to death by targeting the Kv2.1 channel.

### Exploring the expression of KCNB1 in metastases and primary cancerous biopsies

**Fig. 5:**
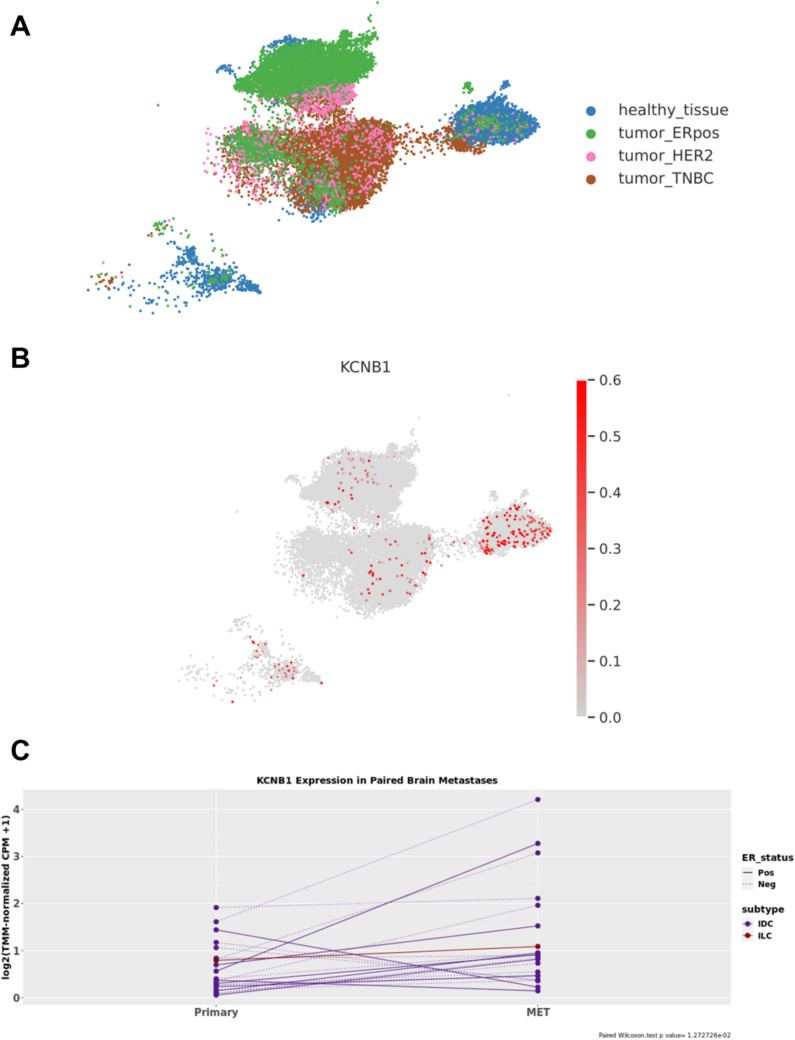
RNA-sequencing of breast cancer subtypes and healthy tissue. (A) the Uniform Manifold Approximation and Projection (UMAP) plot of the clusters deriving from the different breast cancer primary biopsies. (B) the UMAP representing *KCNB1* expression in the single-cell profiles. (C) the *X* axis reports the paired plot of primary breast cancers to brain metastasis (MET), while in Y, *KCNB1* expression is normalized using log2(trimmed mean of M-values copy per million + 1). IDC, invasive ductal carcinome, ILC invasive-lobular carcinoma. Dotted lines indicate ER^−^, while solid lines indicate ER^+^ tumors

We used a RNA-sequencing dataset to evaluate the expression of *KCNB1*, encoding the α-subunit of the Kv2.1 channel. Using two GEO datasets (GSE161529, GSE245601) we well-defined independent clusters containing cells derived from biopsies of different breast cancer subtypes and healthy tissues (Fig. 5A). Concerning the *KCNB1* transcript, we observed its expression in the normal cells (Fig. 5B, corresponding to Bue cells in A), ER^+^ tumors (Fig. 5B, corresponding to Green cells in A) and in TNBCs (Fig. 5B, corresponding to Brown cells in A).

Since the previously considered MDA-MB-436 cells have a metastatic origin, to investigate the putative role of the Kv2.1 channel in metastasis, we analysed another single-cell dataset, employing the webapp from Gene Expression in Metastases Projects (https://shiny.crc.pitt.edu/rnaseq_mets/, last accessed on 10/04/2025) that includes biopsies from 22 brain metastases, 11 bone metastases, 14 ovarian metastases, and 7 gastro-intestinal metastases paired to primary tumors. The *KCNB1* expression is significantly enhanced especially in brain metastases compared to the primary counterpart (paired Wilcoxon test *P* value = 0.013) (Fig. 5C), highlighting the potential of this K^+^ voltage-dependent channel in the metastatic profiles.

## Discussion

Breast cancer, particularly TNBC, is difficult to treat, relying on surgery, radiotherapy, and chemotherapy. Additional approaches are eagerly awaited. The possible target is represented by voltage-gated channels that are important regulators of membrane potential and, consequently, homeostatic processes [62]. Among these types of channels, especially Kv1.1, Kv1.3, Kv1.5, Kv2.1, Kv10.1, and Kv11.1 appear interesting. However, some of them can be inactive or overactivated by mutations [63].

We previously demonstrated that in MDA-MB-231 cells, the Kv10.1 channel contributes to the generation of the membrane potential [15]. Although MDA-MB-436 cells share the same TNBC phenotype, they exhibit different malignant features [64]. In these cells, the possible TG2/Kv10.1 interaction was not associated with ion flux and did not alter the membrane potential, suggesting that other channels may be involved.

The characterization of the expressed channels by transcriptomic profiles and their selective inhibition might support the fight against tumors [65], particularly in tumors characterized by high intra-phenotypic heterogeneity, like TNBCs [66]. Therefore, selective inhibition may represent an important tool for the application of personalized therapies and the study of the channel functioning in specific tumor cell models helps to determine the effects of their modulation. This prompted us to investigate which channel was involved in the membrane current in MDA-MB-436 cells. Firstly, we wanted to understand the relevance of K^+^ ions in the membrane current of MDA-MB-436 cells. Also, we excluded K^+^ ions from the intracellular milieu and then we tested the effects by treatment with the nonspecific K^+^ channel inhibitor TEA-Cl. Both these approaches indicated the relevant contribution of potassium current to the membrane ion flow.

Since the presence of the transcripts of K^+^ channels does not guarantee that the corresponding protein is present and functional, we checked the activity of the channels by the patch-clamp technique measuring ion current crossing the membrane in the presence of specific inhibitors. We reduced the number of channel types to analyze based on data present in the literature excluding non-functional channels such as Kv6.3 or channels that are insensitive to TEA-Cl. Moreover, our recording traces suggested excluding channels with inactivating properties, such as Kv4.1 and Kv12.1. For example, the Kv4.1 channel inactivates rapidly during voltage steps of similar duration to those we used, showing current trace pattern as reported by Greenstein et al. [67] and Kim et al*.* [50] that are different from our data displayed in Fig. 2B, or data describing Kv2.1 channel kinetics in other papers [59]. Therefore, we believe the active channel in our cells is not Kv4.1.

Only two types of channels met the required criteria: the BK and the Kv2.1. The first is Ca^2+^-gated, sensitive to voltage and present in nearly every body cell type, with own distinctive roles for each tissue. BK channel plays an important role in cell division and migration and is involved in the development of several tumors, including breast cancer [8, 68–70]. We investigated its possible contribution to the membrane current of MDA-MB-436 cells reducing its open probability with CdCl_2_, a powerful blocker of Ca^2+^ channels, as already demonstrated [9]. In our experiments, CdCl_2_ was ineffective on MDA-MB-436 cells. Also, the experiments with the BK specific blocker, IbTx did not produce change in the membrane current. We concluded that the BK channels were predominantly silent.

Thereby, we identified the involvement of a specific K^+^ voltage-dependent channel using ScTx1 and Dfe, proposing a meaningful contribution of the Kv2.1 channel in the analyzed cells. Nevertheless, they did not cancel the membrane current. We hypothesize that several factors contribute to the incomplete disappearance of the current. It is a common observation that substances inhibiting ion channel rarely achieve complete inhibition. The interaction between an inhibitor and its target channel is typically a dynamic and reversible process governed by chemical equilibrium. As a result, a certain fraction of channels will inevitably remain open for some duration, even when an inhibitor is present [19]. A key factor in our specific preparation is the presence of a persistent mucous layer surrounding the cell membrane, which resists removal by repeated washes. This layer may obstruct the binding of the inhibitor to the channel.

Another reason may be due to a coupling between K^+^ and Cl^−^ channels [71]. While there is not a simple one-to-one interaction where a potassium channel protein directly "opens" or "closes" a chloride channel protein, the functional and physiological interdependence is strong. K^+^ channels regulate the membrane potential, which in turn sets the driving force for chloride ions, and both channel types are often activated together in a coordinated manner to perform functions like cell volume regulation.

Moreover, K^+^ channels regulate ENaC channels. This regulation is crucial for maintaining electrolyte balance, particularly in the kidney, and is a key mechanism in blood pressure control. The relationship is complex and multifaceted, involving both direct and indirect mechanisms [72].

In addition, we cannot exclude the activation of other types of K^+^ channels or the formation of heteromers channel constituted by subunits of Kv2.1 and other Kv channels.

We did not analyze these highly complex issues; however, they represent an interesting area for future research. The knockdown using RNA interference could represent an additional step in defining the involvement of the channel. However, this molecular approach is not effective in silencing target mRNA in all treated cells. Patch-clamp recordings would therefore be performed blindly, without the possibility of knowing whether the analyzed cells had actually been silenced. As a result, the average membrane current recorded would not reflect a complete suppression of the channel in question, but rather a mixture of currents from leak channels, from channels still active in non-silenced cells, and potentially from off-target effects of the siRNAs. Like other molecules, siRNAs may lack specificity and even affect other members of the same family. Consequently, the use of pharmacological inhibitors remains an important tool.

Blocking of this channel has already been suggested to promote anticancer effects [61, 73]. Our interest in Dfe stems from its well-documented ability to primarily inhibit the Kv2.1 K^+^ channel. Although some potential activity on Kv2.2 cannot be entirely ruled out, no evidence has been published to suggest that Dfe affects ion channels outside of the Kv2 family, such as Kv1, Kv3, or sodium and calcium channels. In our experimental model, represented by the MDA-MB-436 breast cancer cell line, expression of *KCNB2*, which encodes the Kv2.2 channel, was not detected, whereas *KCNB1* transcripts, encoding Kv2.1, were abundantly expressed. Analyzing the effects of Dfe on biological processes of these cancer cells, we observed the inhibition of migration and induction of apoptosis. Concerning apoptosis, it has been reported that it is mediated by the activation of TRPV3 channels [74], however MDA-MB-436 cells employed in these experiments expressed *TRPV3* at very low levels (1 reads of TRPV3 versus 223 of KCNB1, coding Kv.2.1) as detected in RNA-sequencing analysis by Ancona et al*.* [34]. Moreover, activation of TRPV3 would produce an outward current [75] resulting in an increased membrane current, exhibit different kinetics and timescale, and require higher concentrations to observe an effect. For this reason, we are confident that the observed effects mediated by Dfe may be attributed to Kv2.1 channel.

While the membrane current in MDA-MB-231 cells was supported by the Kv10.1 channel [15], the Kv2.1 channel appears to contribute to membrane currents in MDA-MB-436 cells, influencing cell growth and motility. This highlights currently unknown aspects and the need to elucidate the molecular mechanisms triggered by Kv2.1 inhibition, including the signaling cascades and pathways driving the observed anticancer effects. A valuable contribution to advancing knowledge in this area could come from proteomic and phosphoproteomic analyses, which may represent key objectives for future studies.

Inhibition of Kv2.1 channel by drugs is controversial, since under physiological conditions it is active in the nervous system as a regulator of neuronal excitability. Therefore, the use of this drug in breast cancer therapy must be carefully evaluated, to make its action targeted on the cancerous tissue. To answer, at least in part, to this problem, we conducted a study on single cells derived from both healthy and breast cancer tissues and their RNA profiles showed the expression of *KCNB1* especially in TNBCs and ER^+^ phenotypes, but also in normal tissue. *KCNB1* expression is significantly higher in metastases, which would therefore be more sensitive to its inhibitors. Since this channel is highly expressed in nervous tissue, we cannot exclude the presence of contaminants in the brain biopsy samples. However, the substantial number of samples analyzed, 22 matched pairs of primary tumor and brain metastasis, encourages us to believe that the observed differences are meaningful.

In the new era of artificial intelligence, virtual screening [76], and the use of automated systems [77], the design, synthesis, assay, and identification of novel compounds could greatly contribute to this goal. In this perspective, Kv2.1 inhibitor analogs could be further developed, and the MDA-MB-436 cell line could be proposed as a useful model for testing.

In addition, preclinical studies suggest a potential therapeutic role for Kv2.1 inhibition in various cancers, including metastatic prostate cancer, where it reduces cell migration (via ScTx1 and siRNA) [27], as well as cervical carcinomas [23], where it modulates cell proliferation and cycle progression (via hanatoxin-1). These findings support the idea that Kv2.1 represents a promising molecular target for tumor-specific therapies, particularly in aggressive and metastatic cancers.

## Conclusions

The discover of Kv2.1 contribution to membrane current in MDA-MB-436 cells provides new insights into the electrophysiological properties of this TNBC model and supports the potential role of voltage-gated K⁺ channels as therapeutic targets in breast cancer. The use of Dfe to inhibit Kv2.1 induced apoptosis and reduced cell migration, suggesting an its functional involvement in tumor progression. Although Kv2.1 is also expressed in normal tissues, KCNB1 showed higher expression in brain metastases compared to primary tumors, even though further validation would be necessary.

These findings strengthen the rationale for developing selective voltage-gated K⁺ channels inhibitors to enhance tumor specificity and highlight the need to elucidate the activated pathways in order to improve our understanding of their relationship with cancer-related features. In this context, MDA-MB-436 cells may serve as a valuable model for preclinical testing and drug screening aimed at personalized therapeutic approaches in aggressive breast cancer subtypes such as TNBC.

## Data Availability

Data will be made available on request.

## Abbreviations

4-AP: 4-Aminopyridine
BK: Big Potassium
CI: Cell Index
Dfe: Drofenine
DMSO: Dimethyl sulfoxide
FBS: Fetal bovine serum
ScTx1: Stromatoxin
TEA-Cl: Tetraethylammonium chloride
TEA-OH: Tetraethylammonium hydroxide
TNBC: Triple-negative breast cancer
